# Tumeur du sein révélée par une métastase gastrique découverte fortuitement

**DOI:** 10.11604/pamj.2016.24.318.10245

**Published:** 2016-08-19

**Authors:** Aziz El Moussaoui, Fadi Assi, Abdeslam Bental

**Affiliations:** 1 Service de Gastroentérologie, Centre Hospitalier d’Abbeville, France

**Keywords:** Cancer du sein, métastase gastrique, carcinome, Breast tumor, gastric metastasis, carcinoma

## Abstract

Les métastases gastriques du cancer du sein sont rares, et leur découverte reste difficile, devant la symptomatologie qui est souvent non spécifique ou même absente. Nous rapportons une observation originale d'un carcinome canalaire du sein révélé par une métastase gastrique découverte de façon fortuite.

## Abstract (en)

The gastric metastasis of breast cancer are rare, and their discovery is difficult, because the symptoms is often unspecific or even absent. We report an original case of ductal carcinoma of the breast revealed by a gastric metastasis discovered incidentally.

## Introduction

La localisation gastrique de métastases est rare. De multiples cancers primitifs peuvent être à l'origine de métastases gastriques. Le cancer du poumon, le cancer du sein, et le mélanome sont les trois cancers les plus fréquemment responsables. Dans le cancer du sein, le carcinome lobulaire infiltrant est le type histologique donnant plus de métastases gastro intestinales. Nous rapportons une observation originale d'un carcinome canalaire infiltrant du sein révélé par une métastase gastrique découverte de façon fortuite.

## Patient et observation

**Figure 1:**
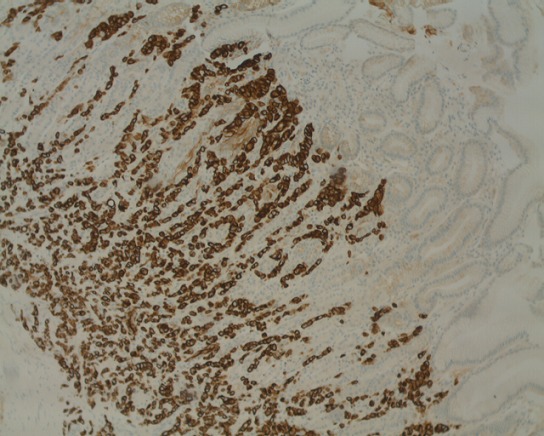
Métastase gastrique d’un carcinome canalaire du sein avec expression des récepteurs cytokératine 7

**Figure 2:**
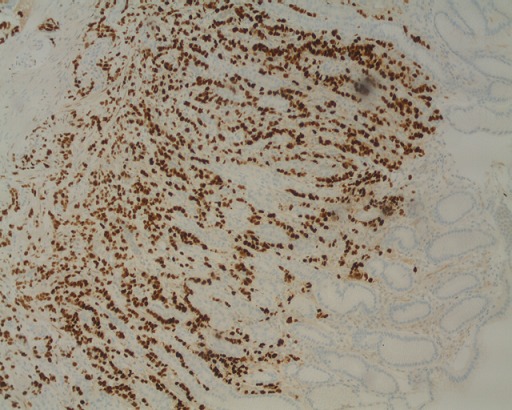
Métastase gastrique d’un carcinome canalaire du sein avec expression des récepteurs oestrogeniques

**Figure 3:**
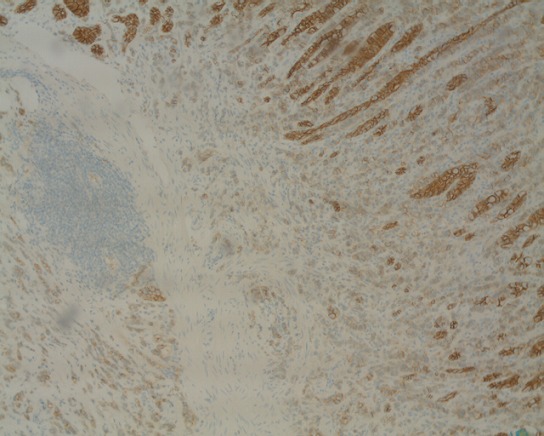
Métastase gastrique d’un carcinome canalaire du sein avec expression des récepteurs E-cadherine

Une patiente âgée de 68 ans, adressée par un hématologue pour une thrombopénie associée à une cytolyse et une cholestase. Une échographie abdominale a été réalisée objectivant un foie de cirrhose. Une fibroscopie oeso- gastroduodénale était faite à la recherche des signes endoscopiques d'hypertension portale, avait objectivé des lésions nodulaires ulcérées au niveau de la grande courbure, tout en précisant que la patiente n'avait aucun symptôme digestif. L'étude anatomo-pathologique couplée à l'étude immuno histochimique a permis de porter le diagnostic d'une métastase d'un carcinome infiltrant à cellules indépendantes du sein, de type canalaire, avec expression des récepteurs pan CK, cK7 (Figure 1), oestrogéniques (Figure 2) et E-cadherine (Figure 3) et absence d'expression des récepteurs ck20, progesteroniques et Her2 neu. La mammographie et l'échographie mammaire mettait en évidence une masse nodulo stellaire située à l'union des quadrants externes du sein gauche. Le bilan d’extension comprenant un scanner thoraco abdomino pelvien, une scintigraphie osseuse, et un scanner cérébral, a montré en plus des métastases gastriques déjà retrouvées à la gastroscopie, des métastases péritonéales, et osseuses. Un traitement à base de Létrozole était instauré.

## Discussion

L'estomac représente un site inhabituel de métastase. Le cancer du sein donne souvent des métastases au niveau de l'os, le poumon et le foie, mais les métastases gastriques restent rares. Dans la série autopsique de Cifuentes N et al, les métastases gastriques sont trouvées dans 10% des cas [1]. Dans une autre étude rétrospective comportant 12001 patientes suivies pour un cancer du sein, seulement 41 ont développé des métastases gastro intestinales, dont 28% siégeait au niveau de l'estomac [2]. La présentation clinique est souvent non spécifique, les métastases gastriques peuvent se manifester par des épigastralgies, des vomissements, ou parfois par une hémorragie digestive. Notre patiente n'avait aucun signe clinique évocateur. Les aspects endoscopiques des métastases gastriques du cancer du sein sont variables. Ils peuvent se présenter sous forme de lésions localisées comme c'est le cas chez notre patiente, d'infiltration diffuse, ou de compression extrinsèque [3].

Sur le plan histologique, le carcinome lobulaire infiltrant du sein est le type le plus souvent incriminé dans les métastases gastriques, alors que notre patiente avait un carcinome canalaire du sein. L'étude immunohistochimique représente un outil d'intérêt majeur, permettant d'orienter le diagnostic (expression des anticorps anti cytokératine 7 et antioestrogene, et absence d'expression des anti cytokératine 20) [4] et de préciser le sous type histologique (expression des récepteurs E-cadherine et p120 pour le carcinome canalaire, et expression unique des récepteurs p120 pour le carcinome lobulaire) [5].

L'utilisation des modalités thérapeutiques combinées (chirurgie, traitement hormonal, chimiothérapie, la radiothérapie), peut permettre d'obtenir une réponse dans 75% des cas [3]. Généralement, la chirurgie est réservée aux situations d'urgence (perforation, hémorragie, et obstruction). Notre patiente était mise sous hormonothérapie. Le pronostic des métastases gastriques est sombre en raison du caractère disséminé de la maladie, avec une survie à 2 ans qui peut atteindre 53% [6].

## Conclusion

Les métastases gastriques constituent un mode de révélation rare du cancer du sein. Leur découverte est souvent tardive ou fortuite, devant la symptomatologie qui reste pauvre et non spécifique.
